# Myocarditis: Differences in Clinical Expression between Patients with ST-Segment Elevation in Electrocardiogram vs. Patients without ST-Segment Elevation

**DOI:** 10.3390/jpm14101057

**Published:** 2024-10-13

**Authors:** Grytė Ramantauskaitė, Kingsley A. Okeke, Vaida Mizarienė

**Affiliations:** 1 Department of Cardiology, Medical Academy, Lithuanian University of Health Sciences, 44307 Kaunas, Lithuania; 2 Medical Academy, Lithuanian University of Health Sciences, 44307 Kaunas, Lithuania

**Keywords:** myocarditis, ST-segment elevation, echocardiography, electrocardiogram

## Abstract

Background/Objectives: In cases of myocarditis, electrocardiograms (ECGs) may suggest a pattern of ST-segment elevation myocardial infarction (STEMI) or non-ST-segment elevation myocardial infarction (NSTEMI). NSTEMI patterns are less frequent in myocarditis cases, but it remains unclear if the presence of ST-segment elevation in myocarditis cases is related to a more severe condition and more damage in the myocardium. Methods: This is a retrospective study involving 38 patients admitted to hospital with myocarditis. Patients were divided into two groups: patients with ST-segment elevation (STE) patterns in the ECG (25), and patients without ST-segment elevation (non-STE) patterns (13). The data compared included results from epidemiological, laboratory, and instrumental tests. Data were analysed using IBM SPSS Statistics v26.0. A *p* value of <0.05 was established as the threshold for statistical significance. Results: C-reactive protein (CRP) levels were higher in the STE group (103.40 ± 82.04 mg/L vs. 43.54 ± 61.93 mg/L, *p* = 0.017). The left ventricle ejection fraction (LVEF) was significantly higher in the non-STE pattern group (49.71 ± 4.14 vs. 56.58 ± 3.99, *p* < 0.001). A lower LVEF correlates with higher TnI levels (r= −0.353, *p* = 0.032) and higher CRP levels (r = −0.554, *p* < 0.001). Lower left ventricle (LV) strain correlates with higher levels of Troponin I (TnI) (r = −0.641, *p* = 0.013). Conclusions: LVEFs in the STE group were lower compared to those in the non-STE pattern group. STE pattern was associated with higher CRP levels. Higher TnI levels in cases of myocarditis were associated with lower LV strain and lower LVEF; higher CRP levels also correlated with lower LVEF. Based on a 6-month echocardiographic follow-up, the prognosis of myocarditis was favourable.

## 1. Introduction

Myocarditis is an inflammatory injury of the myocardium, resulting in symptoms and electrocardiographic changes similar to acute coronary syndromes (ACSs). It is a rare condition that affects approximately 4 to 14 out of every 100,000 people globally each year [1].

Electrocardiograms (ECGs) in cases of myocarditis may suggest patterns of ST-segment elevation myocardial infarction (STEMI) or non-ST-segment elevation myocardial infarction (NSTEMI). Together with elevated blood cardiac troponin concentrations and similar symptoms, it can imitate ACS [2]. ST-segment elevation in the presence of myocarditis presents diagnostic challenges, Although there are certain factors that suggest that a patient may have myocarditis—these patients are typically younger (usually less than 40 years old), have had a recent viral infection, exhibit ECG changes involving more than one vascular territory, and display diffuse or absent wall motion abnormalities on an echocardiogram [3].

The NSTEMI pattern is less frequent in cases of myocarditis [4], but it remains unclear whether the presence of ST-segment elevation in myocarditis is associated with a more severe condition and more damage to the myocardium, even though ST-segment elevation may suggest a more aggressive course of the disease. As there are limited studies evaluating the clinical differences and severity of the myocarditis based on ECG patterns, we aimed to evaluate the differences among myocarditis patients at our centre.

As for future perspectives, studies like this might help predict the course of myocarditis based on ECG patterns and lead to more accurate treatment and monitoring options for these patients. Evaluating the differences between ECG patterns might suggest a more approachable course of management for these patients. Also, there is limited evidence regarding prognostic predictors in cases of myocarditis [5]. With a longer and more standardised follow-up period, ECG patterns may become an important prognostic factor for outcomes.

The objective of this study was to evaluate and compare the epidemiology, laboratory, and instrumental test results of patients with myocarditis and STEMI patterns to those without STEMI patterns.

## 2. Materials and Methods

### 2.1. Study Design and Population

**Figure 1:**
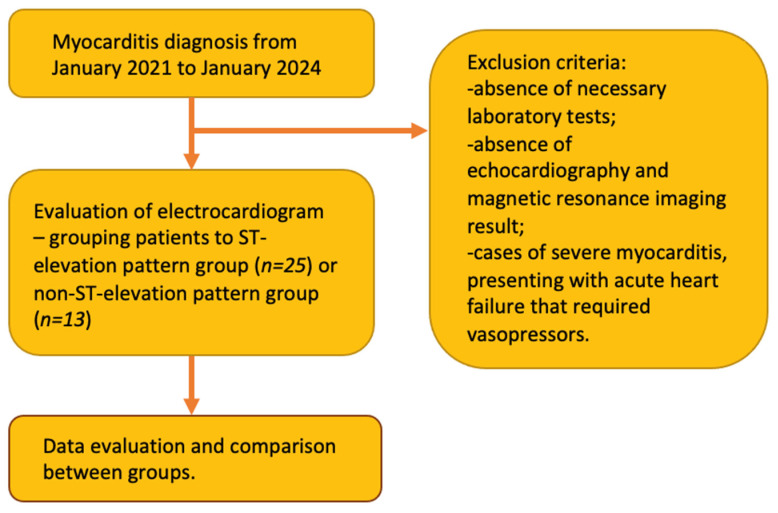
Study design.

This was a retrospective study involving 38 patients who were admitted to our hospital with myocarditis between January 2021 and January 2024. The study design is shown in Figure 1. Patients were enrolled based on the ICD-10 classification codes I40.0, I40.1, I40.8, I40.9, and then evaluated based on the inclusion and exclusion criteria mentioned below. All of the included patients had an ECG performed, as well as laboratory tests that included troponin I (TnI) levels at the time of admission and at the time of discharge, C-reactive protein (CRP) levels at the time of admission and at the time of discharge, creatinine concentration, complete blood count (CBC), and brain natriuretic peptide levels (BNP). Also, all of the included patients underwent echocardiography and cardiac magnetic resonance imaging (MRI) during their hospitalisation. All cases of myocarditis were confirmed by MRI. All of the included patients also underwent a Holter monitoring test. Follow-up echocardiography during the six months after discharge was also evaluated. The methods of treatment were also assessed. Patients were divided into two groups: the first group included patients with an ST-segment elevation pattern on the ECG (25) and the second group included patients without an ST-segment elevation pattern (13).

### 2.2. Inclusion and Exclusion Criteria

Inclusion criteria included being over 18 years of age, a diagnosis of myocarditis in the patient’s clinical history based on ICD-10 classification, and the presence of laboratory tests (TnI, CRP, BNP, creatinine, CBC), echocardiography results, and a diagnosis of myocarditis confirmed by cardiac MRI. Exclusion criteria included the absence of necessary laboratory and instrumental tests, no evidence of myocarditis during cardiac MRI, and cases of severe myocarditis presenting with acute heart failure that required vasopressors. We excluded these patients to focus on evaluating mild and more common forms of myocarditis.

### 2.3. Data Collection

The data were collected retrospectively from medical history that was available through hospital data systems. The data collected included epidemiological data, clinical symptoms, laboratory test results (TnI, CRP, BNP, creatinine concentration, CBC) at the time of admission and at the time of discharge, echocardiographic and heart MRI data during admission, and echocardiographic data at a 6-month follow-up after discharge.

### 2.4. Statistical Data Analysis

Data were analysed using IBM SPSS Statistics v26.0. Data distribution was assessed using a Smirnov–Kolmogorov test; all of the data were distributed normally. Parametric quantitative data were presented as the mean with standard deviation. Parametric comparative data were assessed using Student’s t-test and a paired sample t-test. Numerical data were presented as numbers and percentages. Categorical variables were expressed as absolute numbers (percentages) and compared using the χ^2^ test. Correlations were evaluated using Pearson’s correlation coefficient. A *p* value < 0.05 was established as the threshold for statistical significance.

## 3. Results

### 3.1. Epidemiological and Clinical Differences

Thirty-eight patients were included: twenty-five were assigned to the ST-segment elevation (STE) pattern group and thirteen were assigned to the group without an STE pattern (non-STE pattern group). There were significantly more men in both groups: 96.00% in the STE pattern group and 69.23% in the non-STE pattern group (*p* = 0.038). Patients in the non-STE pattern group were significantly more obese (body mass index (BMI) 25.78 ± 3.95 in the STE group vs. 29.05 ± 5.39, *p* = 0.049). All of the admitted patients had chest pain as a symptom, and the presence of infection (recent or ongoing) was observed significantly more often in the STE pattern group (84.00% vs. 46.15%, *p* = 0.024). All of the epidemiological and clinical differences between the groups are shown in Table 1. All of the patients included in this study underwent a Holter monitoring test and none of these patients were diagnosed with ventricular tachycardia, atrioventricular block, or left/right bundle branch block (*p* = 1.000).

### 3.2. Laboratory Tests Comparison

Laboratory test differences were also assessed; there were higher levels of TnI and BNP in the STE pattern group, but these differences were not statistically significant. There were significantly higher CRP levels in the STE pattern group (103.40 ± 82.04 mg/L vs. 43.54 ± 61.93 mg/L, *p* = 0.017). TnI levels and CRP levels at discharge were also evaluated. There were significantly lower CRP levels at discharge in the non-STE pattern group (12.02 ± 10.82 mg/L vs. 7.10 ± 3.62 mg/L, *p* = 0.002). Other laboratory tests did not differ significantly between the groups. Differences between the laboratory test results are shown in Table 2.

TnI and CRP levels were assessed at admission, the maximal TnI level was measured at that time and again at discharge, and CRP levels were measured at admission and at the time of discharge. There were significantly lower rates of TnI and CRP concentrations at discharge when evaluating all of the study population, regardless of ECG patterns (*p* = 0.010 and *p* < 0.001, accordingly). The results are shown in Table 3.

There was a moderate correlation between higher levels of TnI and higher levels of BNP (r = 0.566, *p* = 0.002). Discharge levels of TnI correlated positively with discharge levels of CRP (r = 0.516, *p* = 0.004), this correlation was moderate.

### 3.3. Echocardiographical Differences

All of the admitted patients underwent echocardiography. Right ventricle (RV) function was normal in all patients. The left ventricle ejection fraction (LVEF) was significantly higher in the non-STE pattern group (49.71 ± 4.14 vs. 56.58 ± 3.99, *p* < 0.001). Other echocardiographic data did not differ significantly between the groups. Echocardiographic differences between the groups are shown in Table 4.

**Figure 2:**
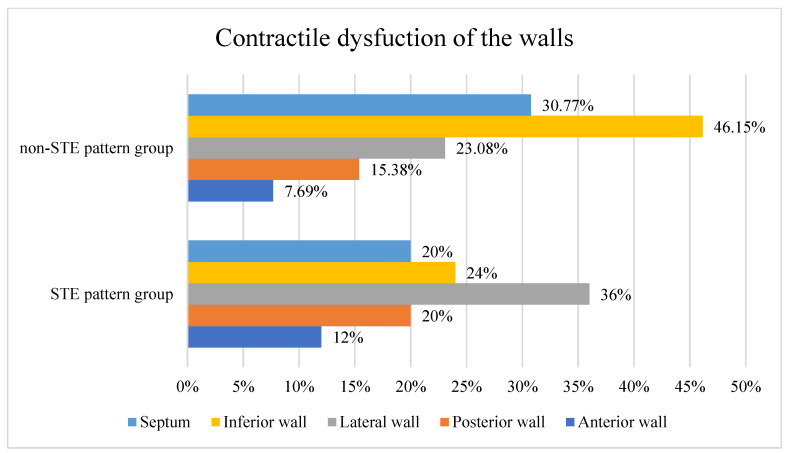
Contractile dysfunction of the walls (*p* = 1.000). Abbreviations: Non-STE—non-STE-segment elevation, STE—ST-segment elevation.

Contractile function was impaired for 9 (69.23%) patients in the non-STE pattern group and for 15 (65.21%) patients in the STE pattern group (*p* = 1.000). The results by location of dysfunction are shown in Figure 2.

Although there were no significant echocardiographic differences between the groups, there were correlations with other parameters. A higher left ventricular end-diastolic diameter (LVEDD) weakly correlated with lower S’(r = −0.361, *p* = 0.033). LVEF correlated with BMI; there was a weak correlation between lower LVEF and higher BMI (r = −0.354, *p* = 0.040). Lower LVEF also correlated with higher TnI levels (r = −0.353, *p* = 0.032), but this correlation was also weak. There was a moderate correlation between lower LVEF and higher CRP levels (r = −0.554, *p* < 0.001). There was a strong correlation between lower left ventricle strain and higher levels of TnI (r = −0.641, *p* = 0.013).

### 3.4. Heart Magnetic Resonance Imaging Differences between the Groups

Heart MRI results did not differ significantly between the groups. Differences between the main parameters are shown in Table 5. In all of the included cases, myocarditis diagnosis was confirmed by heart MRI.

There was a moderate correlation between lower LVEF based on MRI results and a higher TnI concentration (r = −0.479, *p* = 0.003). Also, a moderate correlation was established between higher TnI values and higher left ventricle end-systolic volumes (r = 0.418, *p* = 0.011).

### 3.5. Treatment Differences

Treatment was also compared between groups. The treatment strategy included several options: no specific treatment; only symptomatic treatment (infusions, antipyretics); only antibiotics; optimal heart failure treatment alone; only beta-adrenoblockers; and a combination of antibiotics and optimal heart failure treatment. Significantly more patients in the STE pattern group were prescribed antibiotics and heart failure medications; however, more patients in the non-STE pattern group were prescribed heart failure treatment (*p* = 0.008). The results are shown in Table 6. However, if we take into consideration that both groups of patients (optimal heart failure treatment group and optimal heart failure treatment with antibiotics group) were prescribed heart failure treatment, the results did not differ significantly. Optimal heart failure treatment was prescribed for 9 patients (69.23%) in the non-STE group and 10 patients (40.00%) in the STE group (*p* = 0.170), regardless of antibiotic prescription.

### 3.6. Six-Month Echocardiographical Follow-Up

At the six-month follow-up, echocardiography results were assessed. In the study population, despite the ECG patterns, the LVEF improved during the follow-up, although these data were not statistically significant (52.27 ± 5.59% during admission and 55.45 ± 2.69% at follow-up, *p* = 0.064).

## 4. Discussion

Myocarditis can present in many different clinical forms; it may have an asymptomatic course, cause heart failure (HF) symptoms, or present symptoms of ACS or cardiogenic shock [6]. Most of the patients in our study were admitted to the hospital due to ACS symptoms. All patients had acute chest pain, ECG findings, and laboratory test results similar to those of ACS. We excluded patients who were admitted with cardiogenic shock and treated in an intensive care unit, as we wanted to evaluate only mild and the most common forms of myocarditis. This decision was made by taking into consideration that during the study period, only one patient was admitted to our hospital with myocarditis, severely decreased LVEF, and cardiogenic shock. Including this patient would have disrupted the results, as the other forms included were relatively mild. As reported in the Marburg Myocarditis Registry, which includes a collection of over 1000 patients with documented myocarditis, only 2.5% cases were diagnosed with the fulminant myocarditis phenotype [7]. As this form of myocarditis is rare, we did not have the necessary amount of cases to include those patients in this study. Also, there are not many studies that include only mild cases of myocarditis. According to the American Heart Association’s consensus on the management of acute myocarditis, around 73.4% of reported cases in the recent retrospective registry were mild [8].

In both groups in our study, there were more male patients than female patients diagnosed with myocarditis. These data are consistent with previous studies and registries; according to the literature, myocarditis is more prevalent among men, with male prevalence ranging between 60 and 80% [9]. Moreover, mortality rates in cases of myocarditis are higher among men: 4.4 in every 100,000 cases in women and 6.1 in every 100,000 cases in men worldwide [1]. This may be due to hormonal differences, as hormones are associated with cardiac-immune microenvironments. However, there is a lack of studies which specify the role of sex in the pathogenesis of myocarditis [10].

In patients admitted to the hospital with ACS-like symptoms and test results, it is important to assess ECG changes—whether there is ST-segment elevation or not. Our findings suggest that in myocarditis cases, ST-segment elevation on ECGs predicts more damage to the myocardium—these patients had lower LVEF and higher CRP levels. As previous studies have shown, higher CRP levels correlate with the extent of myocardial damage [11]. Also, patients in the ST-segment elevation group had insignificantly higher TnI levels; higher TnI levels correlated with lower LV strain and lower LVEF, which predict more damage to the myocardium. Multiple clinical studies have demonstrated that an elevation in TnI levels, in the absence of myocardial infarction, impacts long-term prognosis, is associated with cardiovascular related events, and negatively impacts survival [12]. Elevated TnI levels may be considered an important prognostic marker and may lead to more adequate treatment in every single case of myocarditis [13].

In myocarditis cases with an LVEF ≤ 50% and stable haemodynamics, it is recommended to prescribe the usual guideline recommended HF treatment. Beta-blockers should be considered for all patients with myocarditis because of their antiarrhythmic mechanism, which helps prevent ventricular events [9]. Antibiotics are only recommended if there is an active infection of another cause, as myocarditis is usually caused by a viral infection [14]. In our study group, patients in the ST-segment elevation group were prescribed more aggressive treatment. Usually they had signs of other infections and were prescribed antibiotics. There were also more patients with lower LVEF in this group. The prescription of HF treatment alone did not differ between the groups.

In our patient population, none of the patients underwent an endomyocardial biopsy (EMB). This was due to mild forms of myocarditis present; as the American Heart Association/American Heart College recommends, EMB should be performed if a patient is hemodynamically unstable, has high-degree atrioventricular block, or experiences symptomatic ventricular tachycardia [9]. EMB could also be considered in cases of unclear diagnosis [15], as it helps to find the exact cause of the disease and exclude or confirm myocarditis diagnosis and its phenotype. Furthermore, all cases of myocarditis in our study group were confirmed by MRI, so there was no need to confirm diagnosis using interventional methods.

Patients who have myocarditis should avoid sports or intense physical activities for 3 to 6 months after the diagnosis. After this period, patients should undergo follow-up examinations, including echocardiography or MRI. Follow-up echocardiography in our study group showed improved LVEF. These findings led to a good prognosis for these patients. This may be due to the mild–intermediate form of myocarditis in our patient group. Patients with myocarditis may experience partial or full recovery; this relies on several predictors. Poor outcome predictors could include acute HF at onset, ventricular arrhythmias, persistent abnormal levels of TnI, reduced LVEF, and reduced LV strain with no improvement at follow-up [5].

As for future perspectives, studies like this may help predict the course of myocarditis based on ECG patterns and lead to more accurate treatment and monitoring options for these patients from the very beginning of their hospitalisation. Although ECG is a simple and approachable test method, it is often underestimated today. Future findings on the differences between various ECG patterns in myocarditis patients might help to improve the care of these patient and establish more accurate follow-up options. As new methods of evaluating ECGs emerge, such as artificial intelligence, there are many more options to evaluate even subclinical ECG changes, leading to more accurate diagnostics and prediction based on ECG [16].

## 5. Conclusions

In myocarditis cases mimicking ST-segment elevation myocardial infarction, the LVEF is lower compared to patients who had myocarditis without ST-segment elevation in the ECG. Also, ST-segment elevation was associated with higher CRP levels. Higher TnI levels in myocarditis cases were associated with lower LV strain and lower LVEF, higher CRP levels also correlated with lower LVEF. Patients with ST-segment elevation were usually prescribed both heart failure treatment and antibiotics. Based on a 6-month echocardiographic follow-up, the prognosis of myocarditis was favourable.

## 6. Limitations

The limitations of this study include the relatively small study population and possible variations in the echocardiography. As this was a retrospective study, echocardiography was performed based on standardised hospital algorithms. Due to the retrospective nature, there was no ability to standardise the examination protocol for myocarditis patients.

## Figures and Tables

**Table 1:** Epidemiological and clinical differences between the ST-segment elevation (STE) pattern group and the non-ST-segment elevation (non-STE) pattern groups.

	STE Pattern Group (25)	Non-STE Pattern Group (13)	*p* Value
Age (years)	31.88 ± 6.70	30.23 ± 11.28	0.574
Male gender	24 (96.00%)	9 (69.23%)	**0.038**
Female gender	1 (4.00%)	4 (30.77%)	**0.038**
BMI (kg/m^2^)	25.78 ± 3.95	29.05 ± 5.39	**0.049**
Heart rate (bpm)	80.84 ± 13.10	79.00 ± 12.23	0.672
Systolic blood pressure (mmHg)	123.16 ± 13.20	132.85 ± 15.87	0.073
Diastolic blood pressure (mmHg)	76.80 ± 9.36	83.38 ± 7.57	**0.026**
In-hospital days	7.96 ± 3.59	7.08 ± 2.18	0.353
Presence of infection	21 (84.00%)	6 (46.15%)	**0.024**

Abbreviations: BMI—body mass index; non-STE—non-ST-segment elevation; STE—ST-segment elevation. The bolded *p*-values are concerned as statistically significant.

**Table 2:** Differences between the laboratory test results between the STE pattern group and the non-STE pattern group.

	STE Pattern Group (25)	Non-STE Pattern Group (13)	*p* Value
Maximum Troponin I levels (mcg/mL)	15.71 ± 26.72	8.08 ± 7.02	0.200
Troponin I levels at discharge (mcg/mL)	0.32 ± 0.74	0.18 ± 0.31	0.301
CRP at hospitalisation (mg/L)	103.40 ± 82.04	43.54 ± 61.93	**0.017**
CRP at discharge (mg/L)	12.02 ± 10.82	7.10 ± 3.62	**0.002**
Haemoglobin (g/L)	141.16 ± 14.12	143.15 ± 9.34	0.606
Leukocytes (×10^9^/L)	9.35 ± 4.01	8.17 ± 4.19	0.411
Neutrophils (×10^9^/L)	6.85 ± 3.71	5.45 ± 4.05	0.306
Lymphocytes (×10^9^/L)	1.43 ± 0.61	1.61 ± 0.65	0.416
Plasma creatinine concentration (mcmol/L)	75.84 ± 18.42	74.69 ± 12.45	0.822
BNP (ng/L)	75.77 ± 104.18	35.08 ± 38.73	0.144

Abbreviations: BNP—brain natriuretic peptide; CRP—C-reactive protein; non-STE—non-ST-segment elevation; STE—ST-segment elevation. The bolded *p*-values are concerned as statistically significant.

**Table 3:** Differences between Troponin I and C-reactive protein levels at the time of admission and at the time of discharge when evaluating all of the study population, regardless of ECG patterns.

	Mean Concentration at the Time of Admission	Mean Concentration at the Time of Discharge	*p* Value
Troponin I levels (mcg/L)	12.34 ± 24.16	0.27 ± 0.60	**0.010**
CRP levels (mg/L)	82.92 ± 80.24	10.34 ± 9.26	**<0.001**

Abbreviations: CRP—C-reactive protein. The bolded *p*-values are concerned as statistically significant.

**Table 4:** Differences in echocardiography results between the STE pattern group and the non-STE pattern group.

	STE Pattern Group (25)	Non-STE Pattern Group (13)	*p* Value
LVEDD	48.82 ± 3.55	50.69 ± 4.05	0.180
LVEDDi	23.66 ± 1.95	24.00 ± 2.56	0.691
LVMI	88.55 ± 23.03	88.61 ± 19.81	0.994
LVEF	49.71 ± 4.14	56.58 ± 3.99	**<0.001**
LV strain	16.31 ± 3.82	18.93 ± 3.83	0.228
Diastolic dysfunction	7 (28.00%)	3 (23.08%)	1.000
Pericardial effusion	4 (16.00%)	2 (15.38%)	1.000

Abbreviations: LVEDD—left ventricular end-diastolic diameter; LVEDDi—left ventricular end-diastolic diameter index; LVMI—left ventricular myocardial mass index, LV—left ventricle, non-STE—non-ST-segment elevation; STE—ST-segment elevation. The bolded *p*-values are concerned as statistically significant.

**Table 5:** Differences between magnetic resonance imaging (MRI) results between the STE pattern group and the non-STE pattern group.

	STE Pattern Group (25)	Non-STE Pattern Group (13)	*p* Value
LVEF	59.95 ± 5.40	58.23 ± 7.61	0.112
EDV	194.53 ± 32.42	185.51 ± 29.57	0.342
Indexed EDV	94.07 ± 12.72	88.36 ± 10.95	0.682
ESV	77.92 ± 17.80	78.29 ± 22.65	0.158
Indexed ESV	37.84 ± 7.93	37.14 ± 9.14	0.189
SV	116.21 ± 19.74	106.76 ± 15.62	0.945
Indexed SV	60.05 ± 23.09	51.07 ± 7.56	0.811
LVMI	77.07 ± 13.72	74.79 ± 10.65	0.087
Relaxation time T1	1339.53 ± 111.03	1343.69 ± 116.82	0.919
Relaxation time T2	42.74 ± 6.39	46.15 ± 7.04	0.165

Abbreviations: EDV—end-diastolic volume, ESV—end-systolic volume, LVEF—left ventricular ejection fraction, LVMI—left ventricular myocardial mass index, non-STE—non-ST-segment elevation; STE—ST-segment elevation, SV—systolic volume.

**Table 6:** The treatment strategy differences between the STE pattern and the non-STE pattern groups. *p* value = 0.008.

Prescribed Treatment	STE Pattern Group (25)	Non-STE Pattern Group (13)
No specific treatment	7 (28%)	2 (15.38%)
Antibiotics	2 (8%)	0 (0%)
Optimal heart failure treatment	1 (4%)	7 (53.85%)
Beta-adrenoblockers	6 (24%)	2 (15.38%)
Antibiotics and optimal heart failure treatment	9 (36%)	2 (15.38%)

Abbreviations: STE—ST-segment elevation; non-STE—non-ST-segment elevation.

## Data Availability

No additional data were created.
